# Cardiac surgery does not lead to loss of oscillatory components in circulatory signals

**DOI:** 10.14814/phy2.14423

**Published:** 2020-05-06

**Authors:** Kathrine Knai, Petter Aadahl, Nils K. Skjaervold

**Affiliations:** ^1^ Department of Circulation and Medical Imaging Faculty of Medicine and Health Sciences Norwegian University of Science and Technology Trondheim Norway; ^2^ Department of Cardiothoracic Anaesthesia and Intensive Care Clinic of Anaesthesia and Intensive Care Trondheim University Hospital Trondheim Norway

**Keywords:** cardiac surgery patients, circulatory oscillations, continuous blood pressure, electrocardiogram, loss of complexity

## Abstract

The circulatory system is oscillatory in its nature. Oscillatory components linked to physiological processes and underlying regulatory mechanisms are identifiable in circulatory signals. Autonomic regulation is essential for the system's ability to deal with external exposure, and the integrity of oscillations may be considered a hallmark of a healthy system. Loss of complexity is seen as a consequence of several diseases and aging. Heart rate variability is known to decrease after cardiac surgery and remain reduced for up to 6 months. Oscillatory components of circulatory signals are linked to the system's overall complexity. We therefore hypothesize that the frequency distributions of circulatory signals show loss of oscillatory components after cardiac surgery and that the observed changes persist. We investigated the development of the circulatory frequency distributions of eight patients undergoing cardiac surgery by extracting three time series from conventional blood pressure and electrocardiography recordings: systolic blood pressure, heart rate, and amplitude of the electrocardiogram's R‐wave. Four 30‐min selections, representing key events of the perioperative course, were analyzed with the continuous wavelet transform, and average wavelet power spectra illustrated the circulatory frequency distributions. We identified oscillatory components in all patients and variables. Contrary to our hypothesis, they were randomly distributed through frequencies, patients, and situations, thus, not representing any reduction in the overall complexity. One patient showed loss of a 25‐s oscillation after surgery. We present a case where noise is misclassified as an oscillation, raising questions about the robustness of such analyses.

## Graphical abstract

Loss of complexity of biological systems is seen as a consequence of several diseases and aging, and heart rate variability is known to decrease after cardiac surgery. We explore the frequency distributions of ECG and blood pressure recordings of eight patients undergoing cardiac surgery, with the search for loss of oscillatory components and a reduction in overall complexity. Only one patient show loss of a distinct oscillation, and we do not identify any systematic reduction of complexity after cardiac surgery.

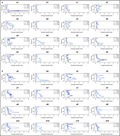

## 1 INTRODUCTION

The circulatory system is oscillatory in its nature. Oscillations can be traced back to physiological processes, such as heart contraction, respiration, and rhythmic contraction of the vasculature, and these oscillatory processes are controlled by regulatory mechanisms. The result is a highly irregular and complex oscillatory profile. Autonomic regulation is essential for the system's ability to deal with external exposure, and the integrity of circulatory oscillations can be considered a hallmark of a healthy system. Circulatory signals, such as continuous blood pressure (BP) and electrocardiography (ECG) signals, show traces of these underlying oscillations. Traditional heart rate variability and frequency analyses have identified specific oscillations and they have been attributed to different parts of autonomic regulation (Akselrod et al., 1981; Bracic & Stefanovska, 1998; Pomeranz et al., 1985). Loss of complexity has been reported in several cardiac and non‐cardiac diseases (Claydon & Krassioukov, 2008; Goldstein et al., 1998; Kleiger, Miller, Bigger, & Moss, 1987; Riordan, Norris, Jenkins, & Morris, 2009; Wolf, Varigos, Hunt, & Sloman, 1978) and simply as a feature of aging (Kaplan et al., 1991; Lipsitz & Goldberger, 1992; Umetani, Singer, McCraty, & Atkinson, 1998; Takahashi et al., 2012). Heart rate variability is known to decrease after cardiac surgery, and remain reduced for up to 6 months (Hogue, Stein, Apostolidou, Lappas, & Kleiger, 1994; Kuo et al., 1999).

There is no clear definition of *complexity*. However, complex systems are built up by components that interact in multiple ways and with the external environment, resulting in organized and disorganized behavior that cannot be predicted from the components alone (Johnson, 2009). Linking this to biological signals, complexity is related to the degree of information in the signal, the predictability of the signal, and the ability to describe the signal in a simple manner (Goldberger, Moody, & Costa, 2012). The definition is too diffuse to provide a quantitative measure of complexity that applies universally. Oscillatory components of biological systems represent underlying components that interact and produce the behavior of the system as a whole. Altogether, they both reflect the system's overall complexity (Goldberger, 1996) and are linked to underlying regulation. On this basis, the exploration of oscillatory distributions of biological signals provides information about the overall state of biological systems, which could be altered by disease or invasive procedures. If the observed changes are generalizable between patients, such information can be implemented to future monitoring tools. This is beneficial as changes in patients’ clinical state could be identified and clinicians notified before overall variables such as heart rate (HR) or BP are changed.

We explore frequency and amplitude modulations of BP and ECG signals by extracting three time series: systolic BP (SBP), HR, and amplitude of the ECG’s R‐wave. The Brody effect states that variations in R‐wave amplitude are related to ventricular preload (Brody, 1956). R‐wave amplitude can thus be seen in relation with SBP and HR. By combining the frequency distributions of the three mentioned variables, we illustrate unique circulatory frequency distributions. In this work, we investigated the development of the circulatory frequency distributions of eight patients undergoing cardiac surgery. Four 30‐min selections, representing key events of the perioperative course, were analyzed with the continuous wavelet transform (CWT), and average wavelet power spectra illustrated the circulatory frequency distributions. We hypothesize that the circulatory frequency distributions show loss of oscillatory components with surgery and that the observed changes persist, measured until the morning after surgery.

## 2 MATERIAL AND METHODS

### 2.1 Study population, ethics, and confidentiality

From March to May 2016, patients scheduled for coronary artery bypass grafting were invited to participate in the study, recruiting a total of 10 patients. Two patients were excluded due to non‐sinus rhythm at one or several time points of the recording. Other exclusion criteria are left ventricular ejection fraction below 0.5, severe valve disease, right ventricular failure, pulmonary hypertension, and severe postoperative hemorrhage. Finally, we had a study group consisting of six men and two women, age ranging from 47 to 88. The patients were enumerated 1–10, with patient 6 and 9 excluded.

The surgery was performed at Trondheim University Hospital, Norway. Written consent was collected prior to data collection. The study protocol was approved by the Regional Committee for Medical and Health Research Ethics (reference: 2015/2019/REK midt). Confidentiality was strictly maintained throughout the study.

### 2.2 Equipment and study protocol

Data collection was performed in two sessions: before and after surgery. The patients were lying in bed during both periods. The study equipment includes a 3‐electrode ECG, a laser Doppler flowmeter (LDF) attached to the calf, and an arterial cannula inserted to the left radial artery. Additionally, patients 1–3 had a photoplethysmograph (PPG) finger sensor attached. The study equipment was provided by ADInstruments (Oxford, UK), as well as hardware and software (PowerLab 16/35 and LabChart 8.1.3). The signals were recorded with a sampling rate of 400 Hz.

The preoperative recordings were collected with the patients resting in bed in a quiet room without disturbances at the thoracic surgery ward. The duration of the recordings ranged from 47 to 86 min. The patients did not receive premedication prior to surgery, and surgery was performed under general balanced anesthesia (thiopental, fentanyl, isoflurane, and propofol). During surgery, the study equipment was removed. After surgery, the study equipment was reattached using new ECG patches and a new arterial cannula inserted to the right radial artery. The postoperative recording was collected from the patients arrived at the thoracic intensive care unit, until the next morning. The duration of the recordings ranged from 14 to 18.5 hr.

### 2.3 Data handling and preprocessing

The BP and ECG recordings were exported from LabChart as mat.files and analyzed in R, version 3.5.1, with the packages *R.matlab, signal, robustHD,* and *WaveletComp* (Alfons, 2016; Bengtsson, 2016; R Foundation for Statistical Computing, 2018; Roesch & Schmidbauer, 2018; Signal developers, 2013). We subdivided the data into four situations: preoperatively (A); postoperatively, on respirator (B); postoperatively, after extubation (C); and postoperatively, the next morning (D).

**FIGURE 1:**
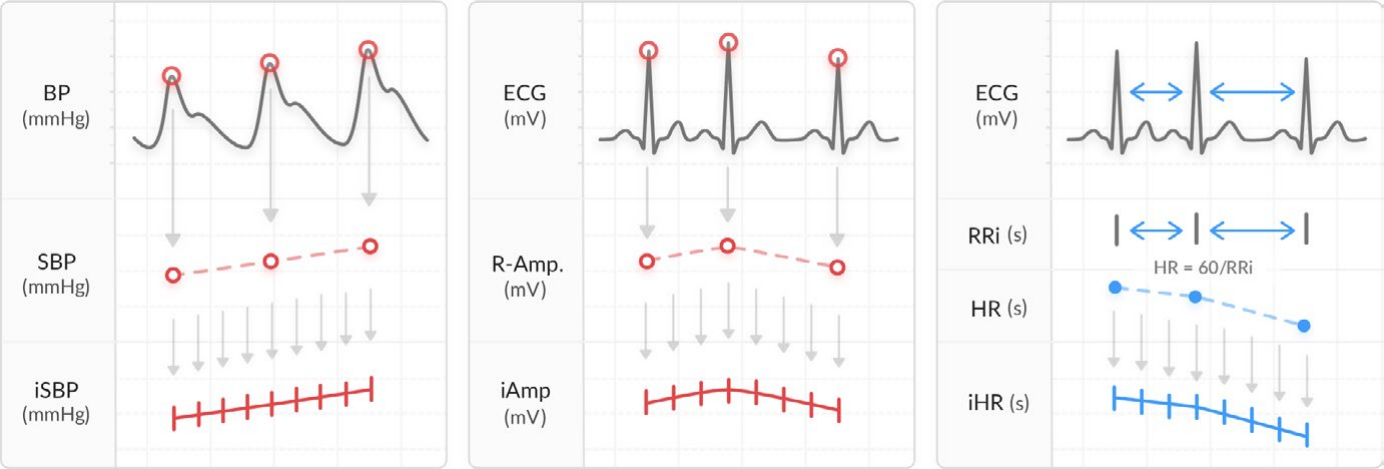
Preprocessing of the BP and ECG signals generating the variables iSBP, iHR, and iAmp. The SBP and the R‐wave amplitude were defined as the maxima of the BP and ECG, respectively. The HR was calculated from the time interval between two subsequent R‐peaks of the ECG (RR_i_ [s]). The time series were interpolated to a sampling rate of 10 Hz

We extracted 30‐min selections representing each situation and preprocessed the BP and ECG signals into three time series: SBP, HR, and R‐wave amplitude (Figure 1). Baseline wander was removed from the ECG signals by applying a Savitzky–Golay smoothing filter before further analyses (Nahiyan & Amin, 2017). We defined the SBP and the R‐wave amplitude as the maxima of the BP and ECG, respectively. The heart rate was defined as HR = 60/RR_i_, where RR_i_ is the time interval in seconds between R‐peak *i* and *i* + 1 of the ECG. Some episodes of noise were misclassified as heartbeats; thus, we removed outliers from SBP, RR‐intervals, and R‐amplitude before further calculation. To provide evenly sampled time series, we performed a cubic spline interpolation to a sampling frequency of 10 Hz. The final variables were called interpolated SBP (iSBP), interpolated HR (iHR), and interpolated R‐wave amplitude (iAmp).

To examine one specific identified oscillation, the PPG and LDF signal of patient 1 were included in a subanalysis. To provide comparable results, the PPG was preprocessed with the same algorithm as iSBP and iAmp, creating a new time series of the amplitude of the signal, interpolated to a sampling rate of 10 Hz. The final variable was called PPG‐iAmp. The LDF was downsampled to 10 Hz.

### 2.4 Analysis

We performed the CWT to identify frequency components present in iSBP, iHR, and iAmp. The CWT is a convolution of the signal with a function generated from the *mother wavelet* (Fugal, 2009). We used the Morlet wavelet, which by mathematical definition is a Gaussian enveloped cosine wave, and has been widely used for investigation of biological signals, especially the ECG (Addison, 2005). In the convolution process, it is shifted in time and stretched and shrunk, quantifying different frequency components’ presence in the signal at different time points. We presented the results in average wavelet power spectra, illustrating the averaged frequency distributions of the signals. Furthermore, we performed the CWT for bivariate time series identifying frequency components that are present in two time series with a significance level of 0.05. The results are presented in cross‐wavelet spectra, with significant frequencies marked by white lines and phase differences by arrows. The CWT for bivariate time series was performed on the variable pairs, iAmp‐iSBP and iSBP‐iHR.

In order to visually examine the individual time‐series’ oscillations, we decomposed the time series with locally weighted estimated scatterplot smoothing (Loess) (Cleveland & Devlin, 1988). We applied the regression three times, each time subtracting the smoothed curve from the signal, providing a set of oscillating components of increasing frequency. The extracted components were called Loess #1, Loess #2, and Loess #3. By plotting the components of all variables together, we visually inspected their oscillating behavior and phase differences. From the CWT and Loess, we identified the components that are highly present in all variables and performed a cross‐correlation analysis, which calculates the correlation of two time series as a function of the displacement of one relative to the other—the cross‐correlation function (CCF). By defining CCF_max_, we identified at which time lag the correlation is highest, and thus at which relative displacement the studied variables oscillate.

## 3 RESULTS

**FIGURE 2:**
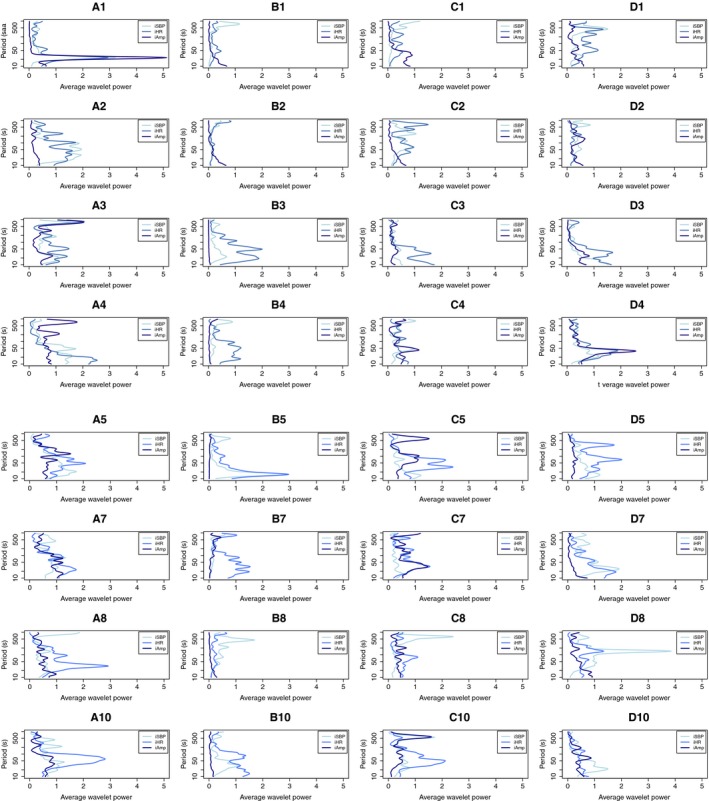
The average wavelet power spectra of iSBP, iHR, and iAmp of all patients through situation A to D. Each spectrum represents one patient in one situation, named with a number and a letter. Patients are separated by rows, and situations by columns. The situations represent key events of the perioperative course: preoperatively (A); postoperatively, on respirator (B); postoperatively, after extubation (C); postoperatively, the next morning (D). Average wavelet power is shown on the *x*‐axis and period (in seconds) on a logarithmic scale on the *y*‐axis. The variables are distinguished by color. Patient 1 shows loss of a 25‐s oscillation between situation A and B. Patient 3 shows loss of an 800‐s oscillation, and patient 8 shows loss of oscillations in the range between 50 and 100 s in iHR

By performing the CWT on iSBP, iHR, and iAmp, we identified each patient's circulatory frequency distribution throughout the perioperative course, illustrated by situation A–D. We hypothesize that we will see loss of oscillatory components from situation A to B, and that the changes persist through situations C and D. Figure 2 shows the average wavelet power spectra of iSBP, iHR, and iAmp of all patients and situations. The spectra include periods between 10 and 1,000 s. Lower periods are not included, as the respiration is a powerful oscillatory component, overshadowing the presence of slower, less dominating oscillations.

We identified oscillatory components in all patients and situations. Patient 1 shows a distinct peak around 25 s in situation A. This oscillation is present in all three variables, and not visible in any of the situations B, C, or D. A less prominent oscillation around 800 s in 3A is observed, which arguably disappears after surgery. Patient 8 showed loss of oscillations in the range between 20 and 50 s in iHR, and in situation D, an oscillation just above 100 s is observed in all variables. We see other examples of loss of power in the mid‐range after surgery, but no cases with loss of distinct oscillatory components. Altogether, we illustrate frequency distributions that change through the perioperative course, but the observed changes do not display any trend or system. Overall, the number of oscillatory components and their power are more or less randomly distributed through patients and situations. Linking this to the signals’ overall complexity, we did not identify any clear reduction of such after surgery. iAmp does not show any distinct oscillatory peaks in any patients in situation B. This is due to a domination of the respiration during mechanical ventilation.

**FIGURE 3:**
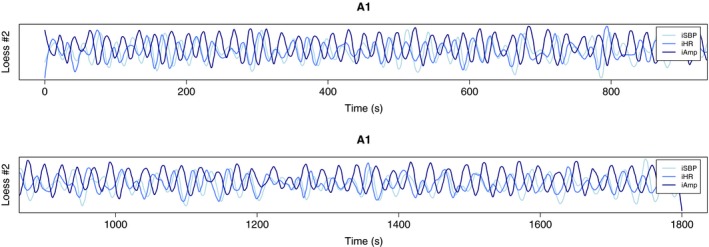
Loess regression of iSBP, iHR, and iAmp of 1A, illustrated by Loess #2 which is the second extracted component. We see that iSBP and iHR oscillates in phase, and iAmp off phase

The 25‐s oscillation in 1A stands out as the only oscillation that is clearly present in all variables before surgery and gone after. To examine the specific variables’ oscillatory behavior, we performed a Loess regression, illustrated by Loess #2 in Figure 3.

**FIGURE 4:**
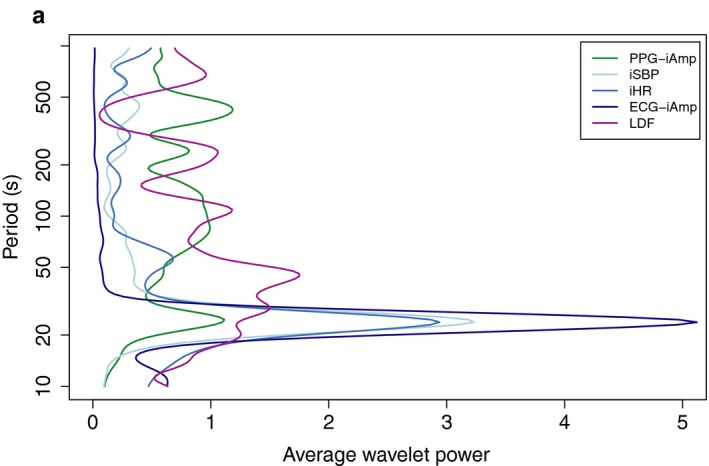
Average wavelet power spectra of 1A including PPG and LDF signals. The PPG is preprocessed with the same algorithm as iSBP and iAmp, giving a time series of the maxima of the PPG signal, called PPG‐iAmp. The LDF is downsampled to a sampling frequency of 10 Hz, as the other time series. The 25‐s oscillation is present in PPG‐iAmp, iSBP, iHR, and ECG‐iAmp, but not in the LDF

Figure 3 shows that iSBP and iHR oscillate in phase, iHR leading. iAmp oscillates off phase with respect to the other two. By performing a cross‐correlation analysis on the variable pairs, iAmp‐iSBP and iSBP‐iHR, we found that CCFmax of iAmp and iSBP is 0.75, with a lag of 8.2 s. The corresponding values for iSBP and iHR are 0.80 and 3.5 s. Altogether, this tells us that iHR is leading, with a time lag of 3.5 s to iSBP and 11.7 s to iAmp. Maximum correlation values of 0.75 and 0.80 are high when it comes to biological time series. The preoperative recording of patient 1 included both a PPG and a LDF signal. The signals were preprocessed as described in Methods and analyzed with the CWT. Figure 4 shows the average wavelet spectrum of all variables, and we see that the 25‐s oscillation is present in the amplitude of the PPG signal but not in LDF.

Figure 2 shows an 800‐s oscillation in patient 3, situation A. It is present in all three variables and is partly gone postoperatively. Looking at the raw signals and performing a Loess regression, we find that the extracted component is caused by short events of noise, thus not representing a true physiological oscillator.

**FIGURE 5:**
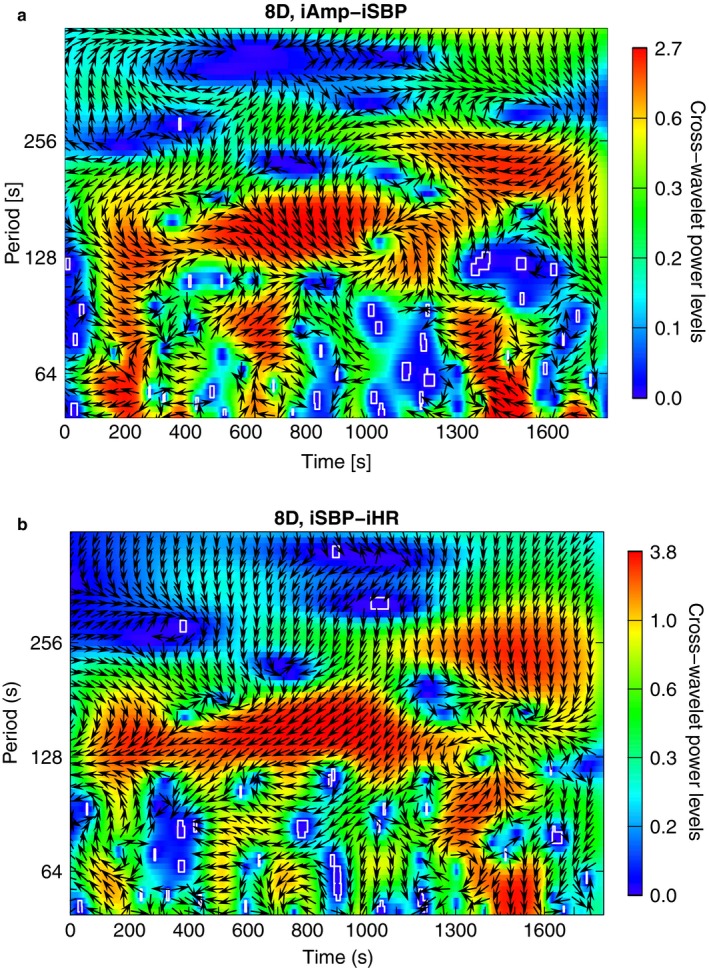
Cross‐wavelet spectra of the variable pairs iAmp‐iSBP and iSBP‐iHR of patient 8, situation D. Time (in seconds) is shown on the *x*‐axes, and period (in seconds) on a logarithmic scale on the *y*‐axes. Power is given by color, according to the scale next to the plots. Significant frequencies are marked by white lines and phase differences by arrows. Both spectra show high power just above 128 s

Patient 8 showed loss of oscillations in the range between 20 and 50 s in iHR, and an oscillation just above 100 s in situation D that is present in all variables. Figure 5 shows the cross‐wavelet spectra of iAmp‐iSBP and iSBP‐iHR of situation D.

Both variable pairs show high power just above 128 s, confirming that the oscillation is present in all three time series. Both variable pairs show variations in phase differences through the time course, but mostly iAmp‐iSBP show arrows pointing to the lower right, meaning that they oscillate in phase, iSBP leading. iSBP‐iHR show arrows pointing to the lower left, meaning that they oscillate off phase, iSBP leading. Thus, iHR and iAmp oscillate in phase, and iSBP out of phase with respect to the other two.

## 4 DISCUSSION

We illustrate the frequency distributions of variables extracted from BP and ECG signals of eight patients undergoing coronary artery bypass grafting. The circulatory frequency distributions illustrate the presence of oscillatory components in all variables, patients and situations, and the oscillations are randomly distributed over the examined frequency range. The high variety seems to represent interindividual variations, more than factors of the performed surgery. Considering the heterogeneity of our findings, we have not presented information that is suitable for use in any monitoring device or other clinical decision tools.

The identified oscillations do not correspond to the distinct pattern of frequency bands that are described in the literature (Akselrod et al., 1981; Bracic & Stefanovska, 1998; Pomeranz et al., 1985). Linking the circulatory frequency distributions to the overall complexity of circulatory signals, no reduction of such is identified. Either the complexity is not reduced with cardiac surgery, or our method is not able to identify it. One case showed a 25‐s oscillation that is present preoperatively (1A) and not postoperatively (1B, 1C, 1D). The oscillation is found in all three variables, and additionally in the amplitude of the PPG (PPG‐iAmp). It has a frequency of 0.04 Hz, corresponding to the limit between low frequencies and very low frequencies (Task Force of the European Society of Cardiology & the North American Society of Pacing & Electrophysiology, 1996). According to the literature, low frequencies reflect baroreceptor activity, but findings are inconsistent regarding whether this activity is mediated by the sympathetic or the parasympathetic nervous system (Shaffer, McCraty, & Zerr, 2014; Task Force of the European Society of Cardiology & the North American Society of Pacing & Electrophysiology, 1996). Patient 8 showed loss of oscillations in iHR with surgery and return of an oscillation in situation D that is present in all variables. The oscillation in 8D has a frequency of 0.007 Hz, corresponding to the ultra‐low frequency band. The physiological correlate to ultra‐low frequencies is insecure. This study was not designed to explore underlying physiological mechanisms of identified oscillations.

We are developing methods for analyzing biological signals, both focusing on preprocessing and choice of analyses. We find that there are several challenges related to analyzing biological signals, mainly related to noise‐handling. We have identified a case where noise is misinterpreted as an oscillation. This leaves us wondering which of the identified oscillations are true physiological oscillators, and which represent methodological errors. As we are not able to provide completely noise‐free recordings in controlled settings of bedbound patients, we believe that tools meant for clinical use must be robust for noise. Thus, the algorithms must either remove all noise, or the results not being affected by the presence of it. We did not identify the distinct frequency bands that are reported in the literature (Shaffer et al., 2014; Task Force of the European Society of Cardiology & the North American Society of Pacing & Electrophysiology, 1996). Noise could be the problem here as well. However, the signals are mostly noise‐free, so we would expect to identify the oscillations if they were present. This raises the question if the use of strict frequency intervals is a simplification of a highly complex and variable field. Features that are incorporated in biological signals, such as nonlinearity and nonstationarity, are challenging when analyzing them. We have earlier addressed the challenge by applying Fourier‐based analyses to such signals, suggesting the data‐driven Hilbert–Huang Transform as a better approach (Knai, Kulia, Molinas, & Skjaervold, 2017). However, the Hilbert–Huang transform is hampered by being computationally challenging and requiring thorough validation. If the developed methods at some time point are meant to be used real‐time, for instance in intelligent alarm systems, the chosen analyses should be quick and easily adaptive to different biological signals. In this work, visual inspection was required to secure that the algorithms applied correctly and to validate the results, identifying the case where noise was misinterpreted as an oscillation.

### 4.1 Methodological considerations

Our study population is small. We recruited 10 patients scheduled for coronary artery bypass grafting over a period of 3 months in 2016, whereof two patients were excluded due to non‐sinus rhythm. The final data material includes three extracted variables from four situations of eight patients—96 analyzed time series. Being derived from only eight patients, we cannot generalize our findings to the total population of cardiac surgery patients.

The recruited patients are heterogeneous individuals featuring different medical backgrounds, pharmacological profiles, and general health. However, the group altogether holds common features such as high age and coronary heart disease. One could raise the question of selection bias as we recruited patients over a short time period and excluded patients with serious illness such as heart failure, valve disease, and perioperative complications. However, we investigate universal physiological features without performing statistical hypothesis testing or other comparisons on group level. The comparisons we do are only between situations of the perioperative course, and in such cases, the patients serve as their own controls. Interpretation of the results must be done with these aspects in thought, and the results’ generalizability should be investigated in bigger study groups.

To minimize autonomic activation and artifacts caused by postural changes, the patients were kept lying during data collection. The data were collected with research hardware and software to secure complete control of filtering and preprocessing algorithms applied to the data. To avoid putting the patients through unnecessary stress by inserting two arterial cannulas prior to surgery, we used different cannulas pre‐ and postoperatively. A consequence of this could be different absolute values of the BP recordings before and after surgery. However, we believe that the frequency distributions of the signals are unchanged. Vasoactive and analgesic medications, and fluids were administered postoperatively according to the individual patient's clinical state. Thus, the patients may have received different amounts of medications, with varying contribution to their oscillatory profile.

## 5 CONCLUSION

In this study, we decomposed BP and ECG recordings from eight cardiac surgery patients to time series of SBP, HR, and R‐wave amplitude. Four 30‐min selections, representing key events of the perioperative course, were analyzed with the CWT and average wavelet power spectra were used to illustrate the patients’ circulatory frequency distributions. We identified oscillatory components in all variables, patients, and situations, and they were more or less randomly distributed through the examined frequency range. The high variety in circulatory oscillations seems to represent interindividual variations, more than factors of the performed surgery. Linking the circulatory frequency distributions to the overall complexity of circulatory signals, no reduction of such is identified. Considering the heterogeneity of our findings, we have not presented information that is suitable for use in any monitoring device or other clinical decision tools. The study is limited by challenges regarding noise‐handling, and generalizability due to small sample size.

## CONFLICT OF INTEREST

All authors declare that they have no competing interests.

## Supporting information



Fig S1‐S2

## References

[phy214423-bib-0001] Addison, P. S. (2005). Wavelet transforms and the ECG: A review. Physiological Measurement, 26(5), R155–R199. 10.1088/0967-3334/26/5/R01 16088052

[phy214423-bib-0002] Akselrod, S. , Gordon, D. , Ubel, F. A. , Shannon, D. C. , Berger, A. C. , & Cohen, R. J. (1981). Power spectrum analysis of heart rate fluctuation: A quantitative probe of beat‐to‐beat cardiovascular control. Science, 213(4504), 220–222. 10.1126/science.6166045 6166045

[phy214423-bib-0003] Alfons, A. . (2016). robustHD: Robust Methods for High‐Dimensional Data [Internet]. Available from: https://CRAN.R-project.org/package=robustHD

[phy214423-bib-0004] Bengtsson, H. (2016). R.matlab: Read and Write MAT Files and Call MATLAB from Within R [Internet]. Available from: https://github.com/HenrikBengtsson/R.matlab

[phy214423-bib-0005] Bracic, M. , & Stefanovska, A. (1998). Wavelet‐based analysis of human blood‐flow dynamics. Bulletin of Mathematical Biology, 60(5), 919–935. 10.1006/bulm.1998.0047 9739620

[phy214423-bib-0006] Brody, D. A. (1956). A theoretical analysis of intracavitary blood mass influence on the heart‐lead relationship. Circulation Research, 4(6), 731–738. 10.1161/01.RES.4.6.731 13365085

[phy214423-bib-0007] Claydon, V. E. , & Krassioukov, A. V. (2008). Clinical correlates of frequency analyses of cardiovascular control after spinal cord injury. American Journal of Physiology‐Heart and Circulatory Physiology, 294(2), H668–H678. 10.1152/ajpheart.00869.2007 18024546

[phy214423-bib-0008] Cleveland, W. S. , & Devlin, S. J. (1988). Locally weighted regression: An approach to regression analysis by local fitting. Journal of the American Statistical Association, 83(403), 596–610. 10.1080/01621459.1988.10478639

[phy214423-bib-0009] Fugal, D. L. (2009). Conceptual wavelets in digital signal processing: An in‐depth, practical approach for the non‐mathematician (p. 382). Space & Signals Technical Pub.

[phy214423-bib-0010] Goldberger, A. L. (1996). Non‐linear dynamics for clinicians: Chaos theory, fractals, and complexity at the bedside. The Lancet, 347(9011), 1312–1314. 10.1016/S0140-6736(96)90948-4 8622511

[phy214423-bib-0011] Goldberger, A. L. , Moody, G. B. , & Costa, M. D. (2012). Variability vs. Complexity [Internet]. Physionet.org. Available from: https://archive.physionet.org/tutorials/cv/

[phy214423-bib-0012] Goldstein, B. , Fiser, D. H. , Kelly, M. M. , Mickelsen, D. , Ruttimann, U. , & Pollack, M. M. (1998). Decomplexification in critical illness and injury: Relationship between heart rate variability, severity of illness, and outcome. Critical Care Medicine, 26(2), 352–357. 10.1097/00003246-199802000-00040 9468175

[phy214423-bib-0013] Hogue, C. W. , Stein, P. K. , Apostolidou, I. , Lappas, D. G. , & Kleiger, R. E. (1994). Alterations in temporal patterns of heart rate variability after coronary artery bypass graft surgery. Anesthesiology, 81(6), 1356–1364. 10.1097/00000542-199412000-00009 7992903

[phy214423-bib-0014] Johnson, N. (2009). Simply complexity: A clear guide to complexity theory (p. 202). Oxford, UK: Oneworld Publications.

[phy214423-bib-0015] Kaplan, D. T. , Furman, M. I. , Pincus, S. M. , Ryan, S. M. , Lipsitz, L. A. , & Goldberger, A. L. (1991). Aging and the complexity of cardiovascular dynamics. Biophysical Journal, 59(4), 945–949. 10.1016/S0006-3495(91)82309-8 2065195 PMC1281262

[phy214423-bib-0016] Kleiger, R. E. , Miller, J. P. , Bigger, J. T. , & Moss, A. J. (1987). Decreased heart rate variability and its association with increased mortality after acute myocardial infarction. The American Journal of Cardiology, 59(4), 256–262. 10.1016/0002-9149(87)90795-8 3812275

[phy214423-bib-0017] Knai, K. , Kulia, G. , Molinas, M. , & Skjaervold, N. K. (2017). Instantaneous frequencies of continuous blood pressure a comparison of the power spectrum, the continuous wavelet transform and the Hilbert‐Huang transform. Advances in Data Science and Adaptive Analysis, 09(04), 1750009.

[phy214423-bib-0018] Kuo, C.‐D. , Chen, G.‐Y. , Lai, S.‐T. , Wang, Y.‐Y. , Shih, C.‐C. , & Wang, J.‐H. (1999). Sequential changes in heart rate variability after coronary artery bypass grafting. The American Journal of Cardiology, 83(5), 776–779. 10.1016/S0002-9149(98)00989-8 10080437

[phy214423-bib-0019] Lipsitz, L. A. , & Goldberger, A. L. (1992). Loss of “complexity” and aging: Potential applications of fractals and chaos theory to senescence. JAMA, 267(13), 1806 10.1001/jama.1992.03480130122036 1482430

[phy214423-bib-0020] Nahiyan, K. M. T. , & Amin, A. A. (2017). Removal of ECG baseline wander using Savitzky‐Golay filter based method. Bangladesh Journal of Medical Physics, 8(1), 32–45. 10.3329/bjmp.v8i1.33932

[phy214423-bib-0021] Pomeranz, B. , Macaulay, R. J. , Caudill, M. A. , Kutz, I. , Adam, D. , Gordon, D. , … Cohen, R. J. . (1985). Assessment of autonomic function in humans by heart rate spectral analysis. American Journal of Physiology‐Heart and Circulatory Physiology, 248(1), H151–H153. 10.1152/ajpheart.1985.248.1.H151 3970172

[phy214423-bib-0022] R Foundation for Statistical Computing . (2018). R: A Language and Environment for Statistical Computing [Internet]. Vienna, Austria: R Foundation for Statistical Computing Available from: https://www.R-project.org

[phy214423-bib-0023] Riordan, W. P. , Norris, P. R. , Jenkins, J. M. , & Morris, J. A. (2009). Early loss of heart rate complexity predicts mortality regardless of mechanism, anatomic location, or severity of injury in 2178 trauma patients. Journal of Surgical Research, 156(2), 283–289. 10.1016/j.jss.2009.03.086 19592027

[phy214423-bib-0024] Roesch, A. , & Schmidbauer, H. (2018). WaveletComp: Computational Wavelet Analysis [Internet]. Available from: https://CRAN.R-project.org/package=WaveletComp

[phy214423-bib-0025] Shaffer, F. , McCraty, R. , & Zerr, C. L. (2014). A healthy heart is not a metronome: An integrative review of the heart's anatomy and heart rate variability. Frontiers in Psychology, 5, 1040. 10.3389/fpsyg.2014.01040 PMC4179748 25324790

[phy214423-bib-0026] Signal developers . (2013). signal: Signal processing [Internet]. Available from: http://r-forge.r-project.org/projects/signal/

[phy214423-bib-0027] Takahashi, A. C. M. , Porta, A. , Melo, R. C. , Quitério, R. J. , da Silva, E. , Borghi‐Silva, A. , … Catai, A. M. . (2012). Aging reduces complexity of heart rate variability assessed by conditional entropy and symbolic analysis. Internal and Emergency Medicine, 7(3), 229–235. 10.1007/s11739-011-0512-z 21253879

[phy214423-bib-0028] Task Force of the European Society of Cardiology and the North American Society of Pacing and Electrophysiology . (1996). Heart rate variability standards of measurement, physiological interpretation, and clinical use. Circulation, 93(5), 1043–1065. 8598068

[phy214423-bib-0029] Umetani, K. , Singer, D. H. , McCraty, R. , & Atkinson, M. (1998). Twenty‐four hour time domain heart rate variability and heart rate: Relations to age and gender over nine decades. Journal of the American College of Cardiology, 31(3), 593–601. 10.1016/S0735-1097(97)00554-8 9502641

[phy214423-bib-0030] Wolf, M. M. , Varigos, G. A. , Hunt, D. , & Sloman, J. G. (1978). Sinus arrhythmia in acute myocardial infarction. Medical Journal of Australia, 2(2), 52–53. 713911 10.5694/j.1326-5377.1978.tb131339.x

