# Supplementary material for: Cardiac surgery does not lead to loss of oscillatory components in circulatory signals
Source: Physiol Rep. 2020 May 6;8(9):e14423. doi: 10.14814/phy2.14423 (PMC7202984; doi:10.14814/phy2.14423)
Supplement: Supplementary file 1 — Fig S1‐S2 [file PHY2-8-e14423-s001.docx]

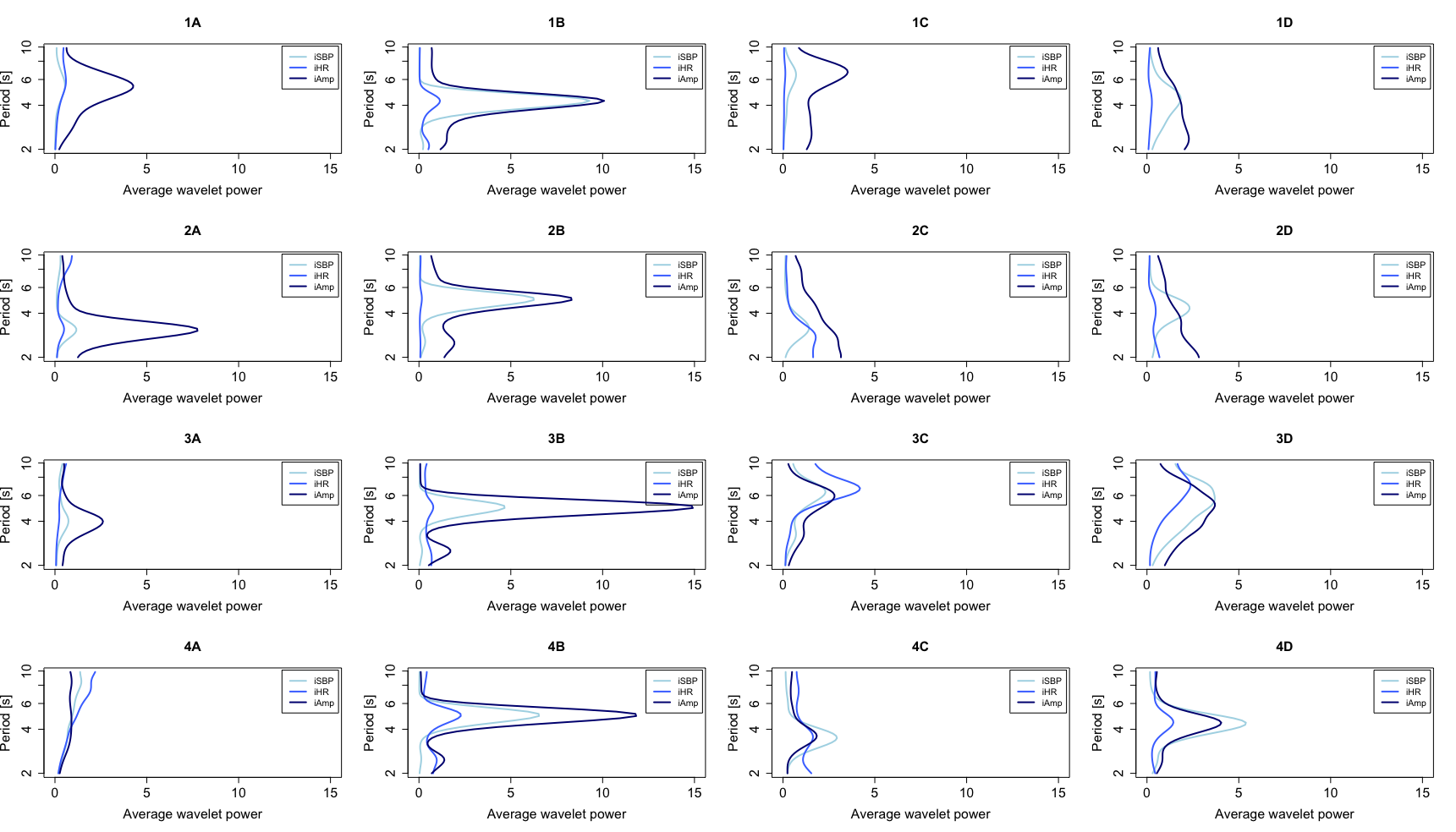


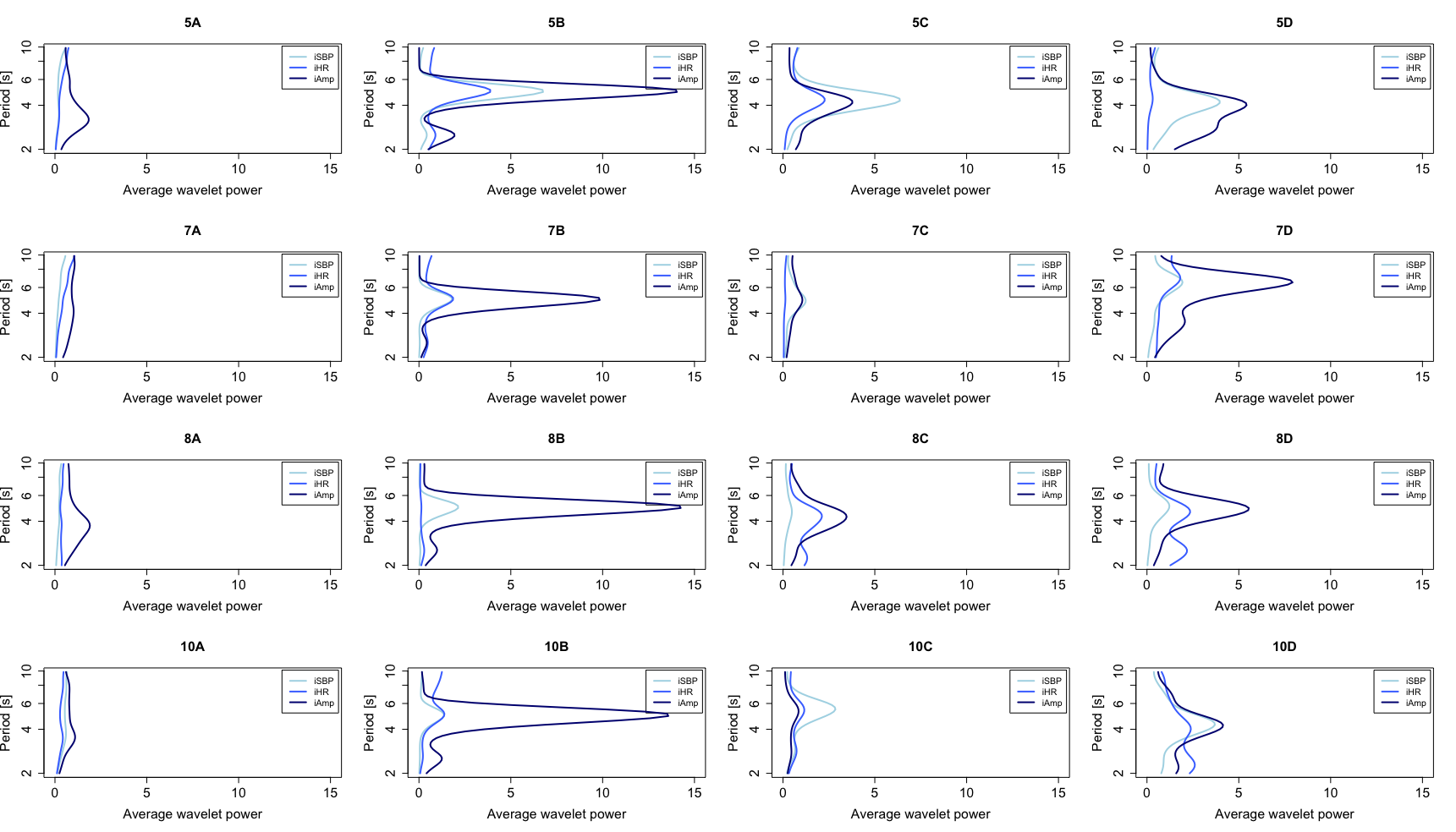


**Supplementary figure 1. Average wavelet power spectra of the respiration**The average wavelet power spectra of iSBP, iHR and iAmp of all patients through situation A to D. Each spectrum represents one patient in one situation, named with a number and a letter. Patients are separated by rows, and situations by columns. The situations represent key events of the perioperative course: preoperatively (A); postoperatively, on respirator (B); postoperatively, after extubation (C); postoperatively, the next morning (D). Average wavelet power is shown on the x-axis and period (in seconds) on a logarithmic scale on the y-axis. The variables are distinguished by colour. We see prominent respiratory components at 5 seconds in situation B, where the patients are mechanically ventilated. Several cases show an additional peak around 2.5 seconds, probably representing a harmonic.

**A**


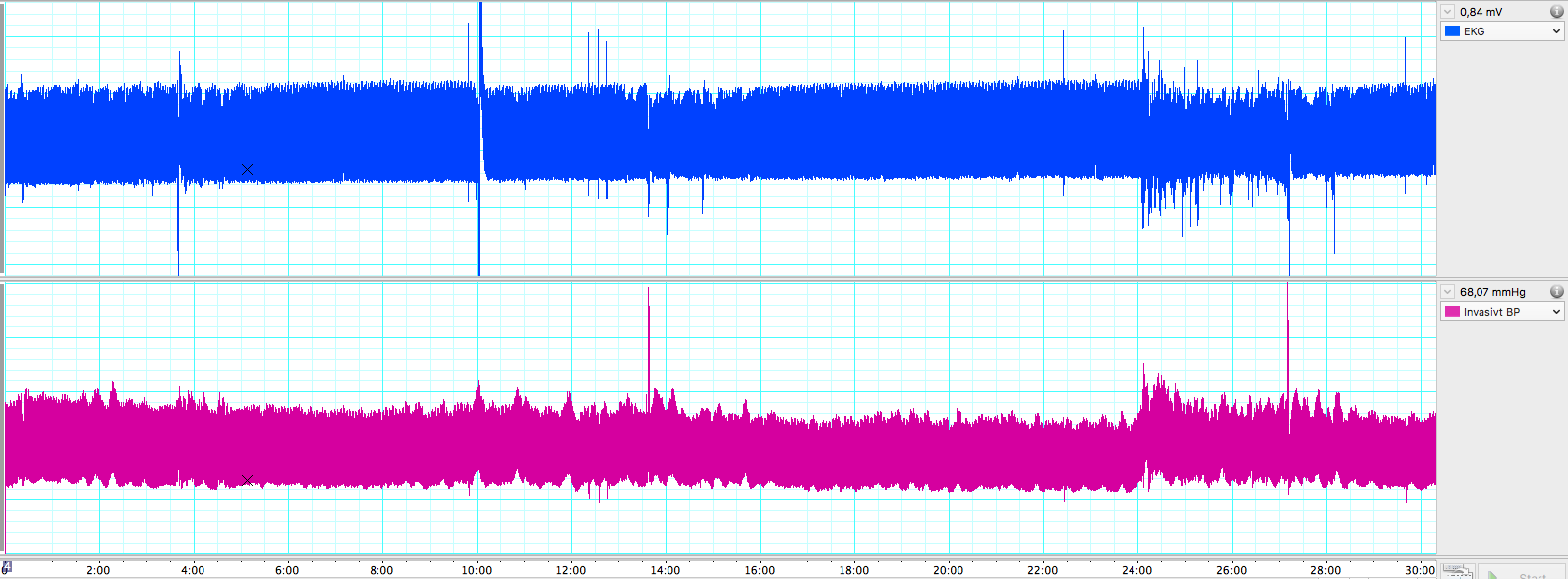


**B**


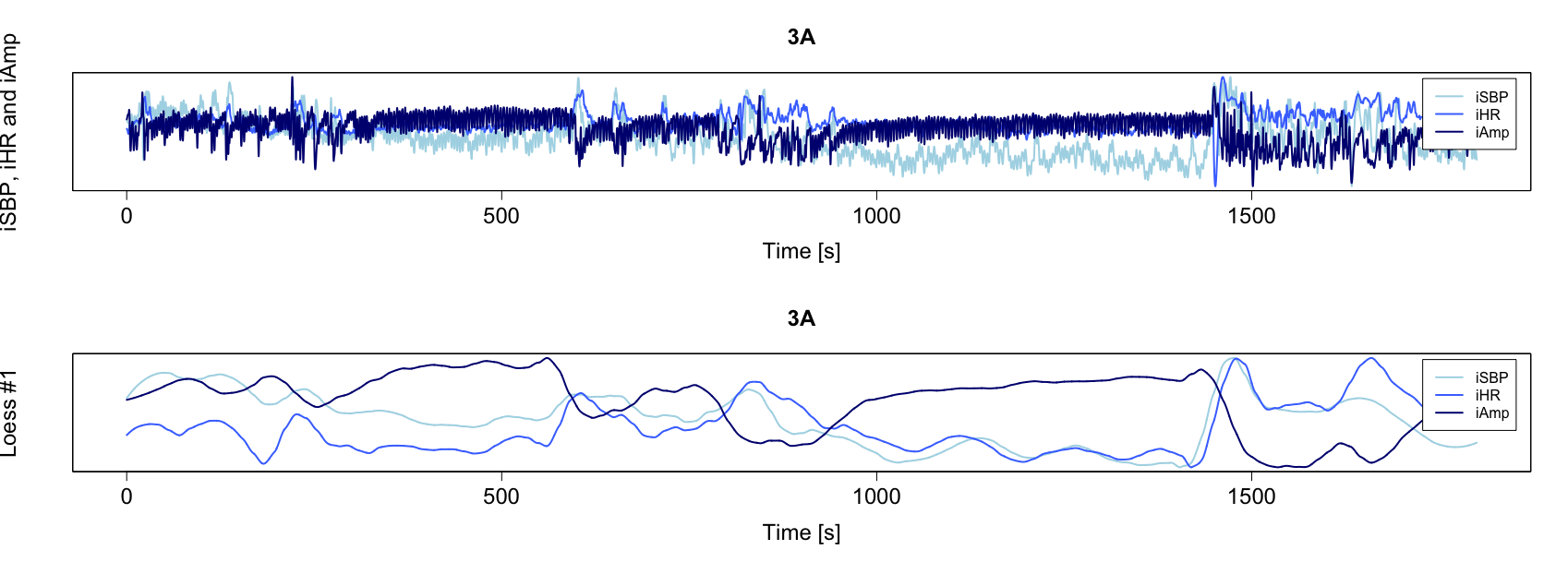


**Supplementary figure 2. Raw signals and Loess #1 of iSBP, iHR and iAmp of 3A**Raw ECG and BP recordings (A) and Loess regression of iSBP, iHR and iAmp (B) of 3A. The 800-seconds oscillation identified with the CWT is caused by short events of noise, giving oscillatory behaviour in all extracted variables. We see from Loess #1 that baseline variations of the variables correspond to noise in the raw signals.
